# A Potential Role for Mitochondrial DNA in the Activation of Oxidative Stress and Inflammation in Liver Disease

**DOI:** 10.1155/2020/5835910

**Published:** 2020-11-16

**Authors:** Wei Xuan, Dandan Song, Youyou Yan, Ming Yang, Yan Sun

**Affiliations:** ^1^Department of Hepatopancreaticobiliary Surgery, China-Japan Union Hospital of Jilin University, Changchun 130033, China; ^2^Department of Clinical Laboratory, Second Hospital of Jilin University, No. 218 Ziqiang Street, Changchun 130041, China; ^3^State Key Laboratory of Inorganic Synthesis and Preparative Chemistry, College of Chemistry, Jilin University, Changchun 130012, China; ^4^Department of Cardiology, Second Hospital of Jilin University, No. 218 Ziqiang Street, Changchun 130041, China; ^5^Department of Molecular Biology, College of Basic Medical Sciences, No. 126 Xinmin Street, Changchun 130041, China; ^6^Department of Anesthesiology, China-Japan Union Hospital of Jilin University, Changchun 130033, China

## Abstract

Mitochondria are organelles that are essential for cellular homeostasis including energy harvesting through oxidative phosphorylation. Mitochondrial dysfunction plays a vital role in liver diseases as it produces a large amount of reactive oxygen species (ROS), in turn leading to further oxidative damage to the structure and function of mitochondria and other cellular components. More severe oxidative damage occurred in mitochondrial DNA (mtDNA) than in nuclear DNA. mtDNA dysfunction results in further oxidative damage as it participates in encoding respiratory chain polypeptides. In addition, mtDNA can leave the mitochondria and enter the cytoplasm and extracellular environment. mtDNA is derived from ancient bacteria, contains many unmethylated CpG dinucleotide repeats similar to bacterial DNA, and thus can induce inflammation to exacerbate damage to liver cells and distal organs by activating toll-like receptor 9, inflammatory bodies, and stimulator of interferon genes (STING). In this review, we focus on the mechanism by which mtDNA alterations cause liver injuries, including nonalcoholic fatty liver, alcoholic liver disease, drug-induced liver injury, viral hepatitis, and liver cancer.

## 1. Introduction

The liver is an organ with high energy requirements, and it plays a key role in the synthesis and secretion of endogenous compounds. Mitochondria are the main source of energy and can use fat- and glucose-derived substrates to generate ATP or energy in the liver [1]. Because the liver needs to continuously synthesize ATP, the density of mitochondria in hepatocytes is relatively higher than that in other cells. Each liver cell contains about 800 mitochondria, which account for approximately 18% of the total hepatocyte volume [2]. Mitochondria also play an important role in liver cell metabolism, are the main sites of fatty acid oxidation and oxidative phosphorylation, play a major role in cell redox homeostasis, and maintain normal liver function [3]. An increasing number of studies have shown that mitochondrial dysfunction plays an important role in the occurrence and development of liver diseases, including nonalcoholic fatty liver, alcoholic liver disease, drug-induced liver injury, viral hepatitis, cholestasis, and liver cancer [4].

### 1.1. The Role of Mitochondrial DNA on Oxidative Stress and Inflammation

Mitochondria are ubiquitous eukaryotic organelles that originated from an ancient *α*-proteobacterium more than 2 billion years ago [5]. Mitochondria are composed of the inner and outer membranes and the matrix and contain their own DNA separated from the nuclear DNA [6]. The outer mitochondrial membrane contains pore protein and multiprotein translocase complex, which allow the passage of molecules. The inner membrane of the mitochondria contains the components for the oxidation reaction of the respiratory chain and ATP synthase [6]. The matrix contains a highly concentrated mixture of hundreds of enzymes, which are involved in the oxidation of pyruvate and fatty acids and the citric acid cycle [6]. Mitochondrial DNA (mtDNA) is a 16569 bp circular double-stranded molecule located in the mitochondrial matrix and contains genes, which specify 2 rRNAs, 22 tRNAs, and 13 polypeptides [7]. All the polypeptides encoded by mtDNA are the components of the oxidative phosphorylation (OXPHOS) system, which is extremely important for energy production [7]. In addition to coding regions, mtDNA also contains a noncoding region, called a replacement loop (D-loop), which controls mtDNA replication and transcription. mtDNA is extremely sensitive to oxidative damage because of its proximity to the inner membrane, lack of protective histones, and the incomplete DNA repair mechanisms in mitochondria [8]. Under physiological conditions, ATP production is mainly dependent on the OXPHOS system, which consists of a series of enzymes located on the inner membrane of the mitochondria, including complexes I (NADH: ubiquinone oxidoreductase), II (succinate dehydrogenase), III (ubiquinol-cytochrome C reductase or cytochrome b), IV (cytochrome C oxidase), and V (ATP synthase) [9]. Mitochondria-derived ROS activate adenosine monophosphate-activated protein kinase (AMPK) and mitogen-activated protein kinases (MAPKs), such as c-Jun N-terminal kinase (JNK) [10]. AMPK stimulates glucose and fatty acid oxidation and activates PGC-1*α* [11]. PGC-1*α* interacts with peroxisome proliferator-activated receptor *α* (PPAR*α*) to induce the expression of several fatty acid metabolic enzymes and increase the mitochondrial *β*-oxidizing acid that acts on fatty acids [12]. In addition, PGC-1*α* also increases mitochondrial transcriptional replication and mitochondrial oxidative phosphorylation [13]. There is growing evidence that mitochondrial dysfunction plays an important role in liver diseases. The accumulation of mtDNA mutations may lead to dysfunction of the mitochondrial respiratory chain, which in turn increases mitochondrial ROS production and subsequent mtDNA mutations. Mitochondrial dysfunction produces a large amount of ROS, which in turn leads to further oxidative damage to the structure and function of mitochondria and other cellular components [14].

In addition to inducing oxidative stress damage, mtDNA contains many unmethylated CpG dinucleotide repeats as it derives from ancient bacteria [5]. When mtDNA leaves the mitochondria and enters the cytoplasm and extracellular environment, it can act as a damage-associated molecular pattern (DAMP) to induce inflammation by activating toll-like receptor 9 (TLR9), inflammatory bodies, and the stimulator of interferon genes (STING) pathway [15]. The inflammation further exacerbates damage to the liver and distal organs. In a mouse model of nonalcoholic steatohepatitis (NASH), mtDNA released from liver cells was found to cause a high level of inflammatory damage [16]. Circulating mtDNA levels are increased in many types of patients, and high TLR9 expression is associated with increased mortality in the intensive care unit [17]. mtDNA also targets the inflammasome, possibly inducing caspase-1 cleavage and activation of interleukin-1*β* (IL-1*β*) and IL-18 [18]. Mitochondria-derived reactive oxygen species (mtROS) enhance the oxidation process and facilitate the cytoplasmic transport of mtDNA after oxidation, and oxidized mtDNA directly binds to NLRP3 to stimulate IL-1*β* production [19]. The activation of NLRP3 inflammatory bodies can further promote the release of mtDNA, and there is a positive feedback loop [19]. TLR/NF-*κ*B activation is a necessary triggering step leading to NLRP3 upregulation and subsequent downstream signaling [20]. The cGAMP synthase- (cGAS-) stimulator of interferon genes (STING) pathway was originally identified as a signaling cascade that is activated by double-stranded DNA (dsDNA) during pathogen infections [21]. Recent studies have shown that mtDNA also activates inflammation via the cGAS-STING pathway, leading to activation of IRF3 and transcription of the type I IFN gene as well as the NF-*κ*B gene [22].

Autophagy is important for mtDNA clearance and can limit the accumulation of proinflammatory factors [23]. Autophagy defects increase caspase-1 activation, IL-1*β* and IL-18 production, and cytoplasmic mtDNA translocation in macrophages induced by LPS and ATP [24]. Phagocytosis of mtDNA by macrophages is another possible clearance mechanism [25]. Moreover, DNases contained in the autophagolysosome play a vital role in degrading mtDNA [26]. For example, knockdown of DNase II amplifies the mtDNA-TLR9-mediated inflammatory response [27].

### 1.2. mtDNA Dysfunction and Nonalcoholic Fatty Liver Disease (NAFLD)

Nonalcoholic fatty liver disease (NAFLD), including liver steatosis, nonalcoholic steatohepatitis (NASH), fibrosis, and cirrhosis, is a disease characterized by excessive accumulation of triglycerides in liver cells and may develop into hepatocellular carcinoma (HCC) or liver failure [28]. NAFLD is closely related to excessive caloric consumption, lack of exercise, insulin resistance, and genetic factors, and its incidence is increasing worldwide. Mitochondrial dysfunction plays an important role in NAFLD as it can decrease energy levels and impair redox balance and reduce the tolerance of hepatocytes to harmful extracellular factors. Different types of mtDNA damage, including deletions, point mutations, and increased 8-hydroxyl levels, have been detected in NASH, indicating that mtDNA dysfunction plays an important role in NAFLD [29, 30].

High-fat, high-sucrose diet can decrease liver mitochondrial content and nicotinamide adenine dinucleotide (NAD+) levels, whereas it can increase oxidative stress in the mouse model of chronic liver sclerosis [31]. Consistently, reduced levels of mitochondrial DNA, oxygen consumption, and ATP production are frequently found in patients who are obese and have type 2 diabetes and in patients with liver steatosis and other liver damage [32]. Electron microscopy analyses have revealed that structure and number of mitochondria are changed and total mtDNA and mtDNA transcription factor A were significantly reduced in the liver of patients with NAFLD [2]. Decreased mtDNA levels can lead to MRC dysfunction and impaired ATP synthesis, increasing the production of ROS, which further cause mtDNA depletion, reducing the number and function of mitochondria [33]. ROS-induced oxidative stress can attack the phospholipid component of the mitochondrial membrane, especially long-chain polyunsaturated fatty acids [34]. In addition, high levels of mtDNA could be released into the plasma in NASH mice with high-fat diet (HFD) [35]. Most of the plasma mtDNA is contained in liver cell-derived microparticles (MPs) and participates in the development of NASH by activating TLR9. TLR9 antagonists prevent the development of NASH, and removal of these MPs from plasma results in a marked reduction in TLR9 activation [35]. HFD-induced mtDNA release can lead to an increase in the chronic sterile inflammatory response by activating the cGAS-cGAMP-STING pathway in mouse adipose tissue [36]. It was favored that STING deficiency partially prevents HFD-induced adipose tissue inflammation, obesity, insulin resistance, and glucose intolerance [37]. mtDNA from hepatocytes of HFD-fed mice was found to induce TNF-*α* and IL-6 expression in cultured Kupffer cells accompanied by activation of NF-*κ*B [38, 39]. STING deficiency or pretreatment with BAY11-7082 (an NF-*κ*B inhibitor) can attenuate the inflammation [38]. HFD-induced obesity and activation of the cGAS-cGAMP-STING pathway are prevented by adipose tissue-specific overexpression of disulfide bond A oxidoreductase-like protein (DsbA-L), a chaperone-like and mitochondrial localized protein whose expression in adipose tissue is considerably suppressed by obesity [40]. Those evidences show that high-fat, high-sucrose diet participates in the development of NAFLD by inducing oxidative stress and causing the release of mtDNA. mtDNA further induces the inflammation to accelerate the liver injury by activation of the TLR9 or cGAS-cGAMP-STING pathway. Therefore, an antioxidant or anti-inflammatory strategy could be used for interfering with the NAFLD.

Inhibition of oxidative stress and inflammation can improve the liver function. The antioxidant MnTBAP (a mimic of manganese superoxide dismutase) can improve the activity of several MRC complexes and improve liver histology in mice [33]. Cardiolipin is a dimeric phospholipid found almost exclusively in the inner mitochondrial membrane (IMM), which plays a key role in mitochondrial oxidative phosphorylation and participates in mitochondrial fusion and division, regulating cell apoptosis [41]. In NAFLD, NR4A1 is upregulated and regulates mitochondrial fission by activating DNA-PKcs and p53 in hepatocytes. p53 promotes mitochondrial fission by enhancing the migration of Drp1 in mitochondria. Blocking the NR4A1/DNA-PKcs/p53 pathway restores mitochondrial phagocytosis and ultimately improves mitochondrial and liver function in NAFLD [42], whereas adding nicotinamide riboside (NAD+ precursor) to the HFHS diet can restore NAFLD by inducing sirtuin (SIRT) 1 and SIRT3-dependent mitochondrial unfolded protein responses, thereby triggering the adaptive mitochondrial pathway to increase liver *β*-oxidation and activity of mitochondrial complexes [31]. Celastrol has recently been identified as a potential new treatment for obesity and can induce liver Sirt1 expression in WT mice in vitro and in vivo [43]. Moreover, celastrol was found to inhibit liver AMP-activated protein kinase *α* (AMPK*α*) and promote the transport of NF-*κ*B to the nucleus, which exacerbates HFD-induced liver damage in LKO mice fed an HFD, resulting in increased liver mRNA levels [43]. Similarly, puerarin can reduce the liver lipid content, inflammation, and fibrosis levels caused by HFHS by restoring NAD+ content via the PARP-1/PI3K/AKT signaling pathway [44]. However, dihydromyricetin (DHM) is the main flavonoid present in rattan tea and has anti-inflammatory and antioxidant properties, as well as hepatoprotective and lipid-regulating effects via induction of SIRT3 and limitation of manganese superoxide dismutase (SOD2) activity [45].

### 1.3. mtDNA Dysfunction and Alcoholic Liver Disease (ALD)

The liver is responsible for metabolizing 90% of ethanol. In liver cells, ethanol is first oxidized to acetaldehyde by alcohol dehydrogenase, then oxidized to acetic acid by acetaldehyde dehydrogenase, and finally metabolized to carbon dioxide and water [46]. Mitochondrial dysfunction is one of the earliest manifestations of alcohol-induced liver damage [47]. Ethanol reduces the mitochondrial membrane potential (MMP) and induces the opening of mitochondrial permeability transition pores (MPTPs), resulting in a decrease in adenosine triphosphate (ATP) synthesis and increased necrosis [47]. High plasma ethanol concentrations can also activate the ethanol oxidation system (MEDS), which catalyzes the production of acetaldehyde. This process increases the consumption of oxygen and NADPH, resulting in hypoxia in cells and increased oxygen free radicals [48]. In addition, acetaldehyde enhances superoxide dismutase (SOD) activity, reduces mitochondrial glutathione, and increases reactive oxygen species (ROS), destroying the antioxidant defense system [49]. Besides induction of oxidative stress, a single dose of ethanol causes massive mtDNA degradation and depletion in mice [50]. Consistently, multiple mtDNA deletions such as those of heteroplasmic 4977, 5385, 5039, and 5556 base pairs were detected in liver tissues from patients with alcohol abuse and severe microvesicular steatosis [51]. In addition, ethanol can cause liver mitochondrial DNA damage such as a specific deletion of about 14.7 kb of mtDNA involving 749-15486 bases, which affects all mtDNA-coding products except the D-loop region [52]. Ethanol enhanced the mtDNA-encoded cytochrome C oxidase (COI) levels, which is associated with enhanced oxidative stress of hepatocytes in a mouse ALD model [53]. mtDNA damage and mROS overproduction caused by excessive mitochondrial fission and defects are involved in the progression of ALD by regulating the orphan nuclear receptor subfamily group 4 A group member 1 (NR4A1)/DNA-PKcs/p53 axis [54]. mtDNA can induce inflammation to accelerate the development of ALD. TNF*α* was increased in alcohol-fed animals and induced the death of liver cells [55]. Alcohol derived the release of mtDNA-containing MPs from hepatocytes, and the mtDNA activated neutrophilia to accelerate the liver injury by activating TLR9 [56]. Another study also showed that mtDNA-enriched MPs promoted inflammation via activation of apoptosis signal-regulating kinase 1 (ASK1) and p38 mitogen-activated protein kinase (p38) [57]. Thus, oxidative stress and mtDNA-mediated inflammation are involved in the development of ALD and might be used as therapeutic targets.

Targeting mitochondria might be used for ALD treatment. Quercetin activates mitochondrial autophagy to inhibit chronic liver mitochondrial damage induced by ethanol in mice by enhancing the expression of Parkin, FOXO3a, and LC3 [58]. Curcumin significantly promotes liver mitochondrial function by reducing the opening of MPTP, thereby increasing MMP and reducing oxidative damage. In addition, curcumin can also reduce the inflammatory response by inhibiting the I*κ*B*α*-NF-*κ*B pathway, thereby reducing the production of TNF, IL-1*β*, and IL-6 [59]. Depletion of mitochondrial glutathione can make liver cells of alcohol-fed animals more sensitive to TNF*α*-induced cell death [55].

### 1.4. mtDNA Dysfunction and Drug-Induced Liver Injury (DILI)

More than 350 drugs have been demonstrated to cause liver injury [60]. DILI includes liver cell lysis, cholestasis, steatosis (i.e., fatty deposits), and steatohepatitis as well as liver cirrhosis and liver cancer [61]. Severe acute fulminant liver failure may require liver transplantation or result in death [62]. Mitochondrial dysfunction plays an important role in DILI [63].

Acetaminophen (APAP) overdose is the leading cause of acute liver failure worldwide. Excessive APAP intake can induce mitochondrial oxidant stress and increase the ROS production and mtDNA damage [64]. It is also found that APAP overdose can rapidly decrease liver mtDNA levels [64]. In addition, mtDNA is released by damaged liver cells and activates neutrophil-mediated inflammation through the binding of TLR9 to further aggravate liver damage. mtDNA/TLR9 in turn induces the microRNA-223 to limit excessive neutrophil activation by targeting NF-*κ*B and liver damage in a negative feedback pathway [65]. Apart from TLR9, reduced mortality and APAP-induced liver injury were found in mice lacking components of the NLRP3 inflammasomes, STING, or cGAS [66]. mtDNA can also activate the NLRP3 inflammasomes capable of recruiting and activating caspase-1, which cleaves pro-IL-1*β* and pro-IL-18 into IL-1*β* and IL-18, respectively [67]. NLRP3 deletion and the related inflammatory body components ASC and caspase-1 could prevent the induction of liver failure [66]. In addition, mtDNA also activate the GAS-STING pathway to accelerate APAP-induced liver injury, and ablation of IFN I recognition in interferon *α*/*β* receptor (IFNAR−/−) mice protected them from APAP-induced liver injury by limiting the GAS-STING pathway [68]. Chlorpromazine is an antischizophrenia dopamine inhibitor that activates autophagy to remove damaged mitochondria and protect mice from APAP-induced liver damage [69]. Thus, mtDNA might be the target of APAP-induced liver damage.

Hepatotoxicity is the most serious side effect of antituberculosis treatment. The isoniazid metabolite hydrazine is a mitochondrial complex II inhibitor. Patients with isoniazid-induced liver injury have more mutations of mtDNA in the NADH subunit 5 and 1 genes of complex I, which may affect respiratory chain function. There is a positive correlation of nonsynonymous mutations in NADH subunit 5 with the ratio of synonymous to total substitution [70]. Linezolid is a particularly effective antibiotic against gram-positive bacteria (*Staphylococcus aureus*, *Enterococcus faecalis*, and *Mycobacterium tuberculosis*) and against resistant pathogens [71]. Severe liver damage was found in some patients treated with linezolid and was correlated with mitochondrial dysfunction [71]. Linezolid inhibits mtDNA translation and reduces the activity of MRC complexes I, III, IV, and V and mtDNA-encoded polypeptides, ND1, and cytochrome C oxidase 2 [71, 72].

Troglitazone derivatives enhance insulin sensitivity through a variety of mechanisms [73]. Troglitazone can cause severe liver damage and was withdrawn from the market in 2006. Troglitazone has strong mitochondrial toxicity and increases the production of ROS in human hepatocytes [74]. Moreover, troglitazone can reduce mtDNA levels by inducing mtDNA strand breaks [74].

Five nucleoside analogs, lamivudine, adefovir, entecavir, telbivudine, tenofovir, and mitochondrial DNA polymerase, have been approved for hepatitis B treatment. These oral antiviral drugs are naturally modified natural nucleoside or nucleotide analogs, have low activity on human mitochondrial DNA (mtDNA) polymerase *γ*, and may cause mitochondrial replication damage and lead to mitochondrial loss or inhibition of function [75]. The seven dideoxynucleoside antiretroviral drugs used to treat human immunodeficiency virus (HIV) infection have been shown to inhibit mitochondrial DNA polymerase, and most of their side effects are the result of their harmful effects on the mitochondrial genome [76]. These drugs can inhibit mtDNA replication, causing profound mtDNA depletion and OXPHOS damage [76]. NRTIs can also induce mtDNA deletion and point mutations, which may be the result of mitochondrial oxidative stress [77].

In addition, some anesthetics also could induce liver injury [78]. For example, isoflurane was reported to induce hepatic dysfunction [79]. Isoflurane induces DNA damage via induction of oxidative stress and inhibition of the repair of DNA damage through the p53 signaling pathway [80]. On the contrary, propofol was also reported to protect the liver against ischemia/reperfusion (I/R) injury by reducing mitochondrial dysfunction, ROS production, and oxidative stress [81]. Halothane induces liver injury, and clinical doses of halothane could decrease the level of mtDNA in an animal model [82].

Those evidences show that a drug induces liver injury via induction of oxidative stress and mtDNA-mediated inflammation. Targeting the mitochondria or mtDNA dysfunction might improve the drug effect.

### 1.5. mtDNA Dysfunction and Viral Hepatitis

Hepatitis B virus (HBV) is an enveloped DNA virus that affects nearly 350 million people worldwide and causes chronic liver disease, liver failure, and hepatocellular carcinoma (HCC) [83]. Hepatitis C virus (HCV) belongs to the Flaviviridae RNA virus family and contains approximately 9400 bp [84]. HCV causes 3-4 million new viral hepatitis cases each year, and about 150 million people are chronically infected; this infection is a risk factor for cirrhosis and/or liver cancer [84]. Mitochondrial dysfunction contributes to HBV-related liver disease [85]. HBV infection can cause Ca^2+^ signal imbalance and increase ROS production, leading to mitochondrial depolarization and dysfunction [83, 86, 87]. Similarly, HCV replication was shown to increase mitochondrial oxidative stress and the production of reactive oxygen species (ROS) [88]. In addition to mitochondria-derived ROS, cellular nicotinamide adenine dinucleotide phosphate (NADPH) oxidase also plays an important role as a source of ROS, generating superoxide anions by catalyzing the oxidation of NADPH in HCV infection [90]. Previous studies have found that HCV infection is associated with decreased mtDNA in hepatocytes [91, 92]. Increased oxidative DNA damage has been found in peripheral blood lymphocytes from patients with HCV infection and in red blood cells in patients with chronic HCV infection [93]. Some studies have shown that HCV genotype 1b is more prone to frequent changes in ultrastructure, high consumption of GSH, decreased mtDNA/nDNA ratio, and increased related lipid peroxidation. mtDNA genetic diversity was found to increase after HCV infection but decrease after HCV clearance, indicating that the effect is reversible depending on the dynamic genetic relationship between HCV and mitochondria [94].

Bicyclol tablets are used to treat chronic hepatitis B and C, and they have been reported to have anti-inflammatory effects on animal models of liver injury [95, 96]. Curcumin is mainly used to treat inflammatory diseases through a variety of mechanisms involving inflammatory transcription factors, cytokines, redox states, or protein kinases [97]. Curcumin can inhibit severe cytokine storms caused by severe viral (such as HIV, HSV, HBV, and HCV) infections [98]. In addition, targeting mitochondrial dysfunction can restore the antiviral activity of exhausted HBV-specific CD8 T cells in chronic hepatitis B [99].

### 1.6. mtDNA Dysfunction and Hepatocellular Carcinoma (HCC)

Hepatocellular carcinoma (HCC) is the most common primary liver cancer and the third leading cause of cancer-related death worldwide [100]. Previous studies have shown that damage to the OXPHOS system and excessive reactive oxygen species (ROS) production are the most important factors for HCC development [101]. Damage to the OXPHOS system and increased ROS levels caused by the destruction of mtDNA can in turn accelerate DNA mutations [101]. This destruction of mtDNA includes point mutations, deletions, insertions, and copy number changes. About 52% of patients with HCC carry at least one homogeneous or heterogeneous point mutation in mtDNA in their tumor tissue. Moreover, 76% of the identified point mutations are located in the D-loop region, 2% are located in the rRNA gene, 3% are located in the tRNA gene, and 19% are located in the gene's mRNA [102]. A 4977-base deletion of mtDNA and a possible relation with gender and long-term drinking history were also found in patients with liver cancer [103]. In one study, among 46 patients with HCC, 30.43% (14/46) of patients had mutations in the D-loop region and 19.57% (9/46) of patients had mutations in MT-ND5 in the D-loop region. The MT-ND5 gene carries the most truncated mutations in colon or rectal adenocarcinomas [104] and is associated with a lack of the mitochondrial respiratory chain [105]. The single-nucleotide repeat sequence is the most susceptible to oxidative damage. This tandem or triple repeat is detected in about 4% of HCCs and is highly correlated with the presence of poly-C length changes at np 568 [106].

A low mtDNA content was found to stimulate NF-*κ*B and to be positively correlated with HCC tumor size [107, 108]. mtDNA can induce inflammation via the STING pathway. Consistently, STING-deficient mice were reported to exhibit an unaltered initial development of HCC but had a higher number of large tumors at the late stages of the disease [109]. Hypoxia induces the intracellular translocation and release of a variety of damage-associated molecular patterns (DAMPs) such as nuclear HMGB1 and mtDNA. HMGB1 binds mtDNA in the cytoplasm of hypoxic tumor cells and promotes tumor growth through activating TLR9 signaling pathways [110]. mtDNA promotes inflammation by activating TLR9. Notably, HMGB1 upregulates mitochondrial biogenesis in HCC cancer cells, promoting tumor survival and proliferation during hypoxia [110]. Dynamin-related protein 1- (Drp1-) mediated mitochondrial fission was found to induce cytosolic mtDNA stress to enhance CCL2 secretion from HCC cells by the TLR9-mediated NF-*κ*B signaling pathway, thus promoting TAM recruitment and polarization [111].

Targeting mitochondria is a promising therapeutic strategy for treating various cancers. The antibiotic tigecycline inhibits proliferation and promotes apoptosis in hepatocellular carcinoma (HCC) by inducing mitochondrial dysfunction and oxidative damage [112]. Tigecycline can specifically inhibit mitochondrial translation, such as by decreasing protein levels of Cox-1 and Cox-2 (but not reducing levels of Cox-4 or Grp78), lowering mitochondrial membrane potential, and increasing ROS levels to induce mitochondrial dysfunction in HCC cells [112]. Consistent with oxidative stress, oxidative damage to DNA, proteins, and lipids has also been observed in tigecycline-treated cells. Importantly, the antioxidant N-acetyl-l-cysteine (NAC) reverses the effect of tigecycline, suggesting that oxidative stress is necessary for the role of tigecycline in HCC cells [113]. Dihydroartemisinin (DHA), an effective antimalarial drug isolated from the traditional Chinese medicine *Artemisia annua*, can inhibit ROS and AIM2/caspase-1 inflammasomes via induction of autophagy in HCC [114]. Autophagy also can reduce mtDNA, which participates in liver injury via induction of ROS and inflammation. Those studies indicate that DHA might regulate the mtDNA-induced oxidative stress and inflammation via induction of autophagy.

## 2. Conclusions

**Figure 1:**
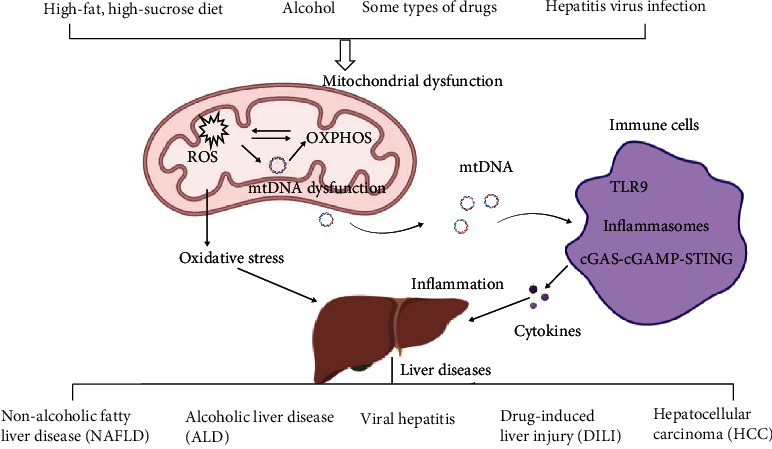
Overview of mtDNA-mediated oxidative stress and inflammation in liver disease. High-fat, high-sucrose diet, alcohol, some types of drug, and hepatitis virus infection can increase the production of ROS and cause mitochondrial dysfunction. Increasing mitochondrial stress induces the release of damaged or fragmented mtDNA into the cytosol or extracellular space. mtDNA can induce inflammation to exacerbate liver injury by activating TLR9, inflammasomes, and cGAS-STING of immune cells.

In this review, we focused on the role of mtDNA dysfunction in liver injuries, including nonalcoholic fatty liver, alcoholic liver disease, drug-induced liver injury, viral hepatitis, and liver cancer. Many types of mtDNA damage, including point mutations, deletions, insertions, and copy number changes, have been found in liver injuries. The mtDNA damage increases ROS and induces liver oxidative stress injury in hepatocytes. Moreover, mtDNA can be released into the cytosolic and extracellular environments, and it can act as damage-associated molecular patterns (DAMPs) to trigger the inflammatory response through TLR9, NLRP3, or STING pathways and further exacerbate hepatocellular damage and even remote organ injury (Figure 1). In conclusion, mtDNA contributes to the pathogenesis of liver injury, and targeting mitochondria is a promising therapeutic strategy for treating liver injuries.
